# MULTIMORBIDITY PATTERNS ARE DIFFERENTLY ASSOCIATED WITH DEPRESSION IN MIDDLE-AGED AND OLDER CHINESE

**DOI:** 10.1093/geroni/igz038.2906

**Published:** 2019-11-08

**Authors:** Shan-Shan Yao, Gui-Ying Cao, Zi-Shuo Chen, Zi-Ting Huang, Beibei Xu

**Affiliations:** 1 Department of Epidemiology and bio-statistics School of Public Health, Peking University, Beijing, China; 2 Peking University, Beijing, China; 3 Peking University Medical Informatics Center, Beijing, China

## Abstract

The associations of multiple physical conditions with depression are still unclear. This study examined the relationship between physical multimorbidity patterns and depression among middle-aged and older Chinese. Patterns of physical multimorbidity were identified using Exploratory Factor Analysis (EFA) among 21,933 participants ≥ 45 years from 2011 to 2015. Multiple logistic regressions were performed to assess the associations between multimorbidity, multimorbidity patterns (factor scores) and depression for each age group (45-60 years vs. ≥60 years). The overall prevalence of multimorbidity was 40% and it was higher among participants with depression (54%) than those without depression (33%). Middle-aged (OR: 1.45; 95%CI 1.16–1.80) and older (OR: 1.85; 95%CI 1.62–2.11) adults with multimorbidity were more likely to have depression compared with those without multimorbidity. Five multimorbidity patterns were identified: cardio-metabolic, respiratory, splanchnic, cardio-cerebrovascular, and tumor-and-degenerative. Middle-aged participants with higher respiratory pattern score had a higher odds to have depression (OR: 1.59; 95%CI 1.15–2.21). Among older adults, higher cardio-metabolic pattern score was significantly associated with lower odds of depression (OR: 0.78; 95% CI 0.63–0.97), while higher respiratory (OR: 1.32; 95%CI 1.04–1.68), splanchnic (OR: 1.22; 95%CI 1.01–1.47) and tumor-and-degenerative pattern scores (OR: 1.86; 95%CI 1.42–2.43) were all found to be significantly associated with higher risk of depression. The associations between physical multimorbidity patterns and depression differ by age. Future studies are needed to investigate the temporal nature of how physical multimorbidity patterns may induce depression and the underlying mechanisms.

